# Luteolin enhances the antitumor efficacy of oncolytic vaccinia virus that harbors IL‐24 gene in liver cancer cells

**DOI:** 10.1002/jcla.23677

**Published:** 2020-12-03

**Authors:** Chunming Wang, Qiang Li, Boduan Xiao, Huiling Fang, Biao Huang, Fang Huang, Yigang Wang

**Affiliations:** ^1^ College of Life Sciences and Medicine Zhejiang Sci‐Tech University Hangzhou China; ^2^ Department of Pathology Zhejiang Provincial People's Hospital People's Hospital of Hangzhou Medical College Hangzhou China

**Keywords:** IL‐24 gene, liver cancer, luteolin, oncolytic vaccinia virus

## Abstract

**Background:**

Interleukin 24 (IL‐24) is an IL‐10 family member and a secreted cytokine characterized by cancer‐targeted toxicity and can activate apoptosis by sensitizing cancer cells to chemotherapy. Cytotoxic effects of luteolin on different types of cancer cells suppress their growth by acting on the components of the apoptosis signaling cascade. Therefore, our study aimed to prove whether oncolytic vaccinia virus (VV) that harbors IL‐24 (VV‐IL‐24) combine with luteolin exerts a synergistic inhibitory effect in liver cancer cells.

**Methods:**

Impacts on cell viability of VV‐IL‐24 and luteolin were assessed by MTT in various liver cancer cell lines. Then, liver cancer cell apoptosis was analyzed via flow cytometry and Western blotting. Besides, the MHCC97‐H xenograft mouse model was employed as a means of assessing in vivo antitumor efficacy.

**Results:**

MTT assay confirmed that the combination treatment decreased liver cancer cells viability to a greater degree than treatment with VV‐IL‐24 or luteolin alone. Flow cytometry and Western blot assay proved that VV‐IL‐24 plus luteolin induced more liver cancer cells apoptosis than single treatment. Furthermore, in the MHCC97‐H xenograft model, 15 days of treatment with VV‐IL‐24 plus luteolin inhibited tumor growth significantly more than single treatment.

**Conclusion:**

These data confirm that the synergistic mechanism of VV‐IL‐24 and luteolin elicits a stronger tumor growth inhibition than any single therapy. Thus, the combination of VV‐IL‐24 and luteolin could provide the basis for preclinical research in the treatment of liver cancer.

## Graphical abstract

The combinational treatment of Oncolyticvaccinia virus VV‐IL‐24 and luteolin resulted in stronger oncolysis and IL‐24 expression compared with VV‐IL‐24 treatment alone, which could promote HCC cell apoptosis and antiangiogenesis effect, suppress tumor cell proliferation.

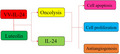

## 1 INTRODUCTION

The pathogenesis of liver cancer involves genetic and epigenetic alterations. Usually, treatments such as surgical resection, local ablation and chemotherapy can only be effective in the early stage, but no therapies are available for advanced liver cancer.^1^ Liver cancer is complex since it involves modifications and cross‐talk among several signal pathways.^2^ Therefore, the development of combination therapy could provide superior treatment for liver cancer in the future.

Vaccinia virus (VV) has a variety of mechanisms for antitumor efficacy, including direct oncolysis, suppression of tumor‐induced immune response, and anti‐angiogenesis.^3^, ^4^ Normal cells have generally low nucleotide concentrations, and thymidine kinase (TK) is linked with deoxyribonucleotide synthesis, which is necessary for DNA replication. However, cancer cells have high concentrations; therefore, TK is dispensable for cancer cells proliferation.^5^, ^6^ Thymidine kinase deleted VVs in many animal models including melanoma and breast carcinoma have shown that VVs replication selected in tumor.^7^, ^8^ A previous study has shown that vaccinia virus encoding IL‐24 can target and kill lung cancer cells without impacting normal cells.^9^ Furthermore, replication‐competent oncolytic vaccine virus (VV) that selectively infect tumors are emerging as an attractive therapeutic target in liver cancer.^10^ Thus, VV may be a good candidate for the systemic oncolytic virotherapy of human tumors.

Prior studies have assessed interleukin 24 (IL‐24) in various cancer cells,^11^ such as colon cancer,^12^ prostate cancer^13^ without exerting significant side effects on normal cells. The strong expression of IL‐24 induces the promotion of tumor cell apoptosis which inhibits tumor growth, angiogenesis, or metastasis and even enhances immunoregulation.^14^ The anticancer effect of IL‐24 also includes the bystander effect killing nearby tumor cells.^15^ To improve the therapeutic efficacy of IL‐24, we searched for a synergistically acting antitumor drug that could be employed together with IL‐24 in combination therapy.

Luteolin (3′,4′,5,7‐tetrahydroxyflavone) can be obtained from various fruits and vegetables,^16^ and is a flavonoid. Luteolin suppresses the growth of cell lines derived from different types of tumors, and this effect is mediated by the promotion of apoptosis and inhibition of proliferation.^17^ The mechanism of the activation of liver cancer cells apoptosis by luteolin induces mitochondria translocation of Bax/Bak and the stimulation of JNK signaling.^18^ Besides, luteolin enhances the expression of oncolytic adenovirus‐mediated E1A and TRAIL proteins, which leads to augment antitumor effects by enhancing colorectal cancer cells apoptosis.^19^

In this present study, the combination of VV‐IL‐24 and luteolin markedly decreased viability and significantly increased liver cancer cells apoptosis. The mechanism was explored to provide a novel strategy on the apply of VV‐IL‐24 and luteolin in the therapy of liver cancer.

## 2 MATERIALS AND METHODS

### 2.1 Cell lines and reagents

The human normal liver cells LO2, the human MHCC97‐H, HepG2, PLC/PRF/5, Hep3B liver cancer cell lines, and HEK293 were purchased from the Type Culture Collection of the Chinese Academy of Sciences (Shanghai, China). All cells were grown in DMEM supplemented with 10% FBS (all from Gibco, MA, USA) in a humidified incubator at 5% CO_2_ and 37°C Luteolin was purchased from Beyotime Institute of Biotechnology, Haimen, China, and stored at 4°C. Mycoplasma negativity was confirmed in all utilized cell lines.

### 2.2 Construction and purification of VV‐IL‐24

An expression cassette under the control of the viral p‐se/l and p‐7.5 k promoters encoding the IL‐24 and gpt genes was used to construct VV‐IL‐24 via insertion into the region of the viral TK gene, as previously described.^9^ Viral amplification was conducted via the infection of HEK293 cells, with tissue culture infectious dose (TCID50) assay being used for tittering in HEK293 cells.

### 2.3 MTT assay

Cells (5,000/well) were added to 96‐well plates. The EC50 concentration of luteolin in normal liver cell LO2 for 48 h was 11.96 µg/ml, which was detected by us. As a result, 5 μg/ml luteolin was considered as a safe dose and used in the following experiments. Then, the normal liver cells and liver cancer cells were infected using VV‐IL‐24 (4 MOI) and luteolin (5 µg/ml) or both VV‐IL‐24 (4 MOI) and luteolin (5 µg/ml). Cells were incubated for 24, 48, 72 or 96 h after which 20 µl MTT (Macklin) was added per well for 4 h at 37°C. Supernatants were then removed, and 150 µl DMSO (Amresco) was added per well. Plates were agitated for 10 min, after which absorbance at 490 nm was assessed via Microplate Reader (Tecan Group, Ltd., Mannedorf, Switzerland). CalcuSyn v2.1 (Biosoft, Cambridge, UK) was used to calculate combination index (CI) values, and average CIs from three independent experiments were designed as X‐mark on the graph. when CI = 1 indicates an additive relationship, CI > 1 indicates antagonism, and CI < 1 indicates synergy.^20^

### 2.4 Flow cytometric analysis

Cells (5 × 10^5^) were added to 6‐well plates per well. Then, liver cancer cells were then subjected to treatment with VV‐IL‐24 (4 MOI) and luteolin (5 µg/ml) or both VV‐IL‐24 (4 MOI) and luteolin (5 µg/ml). Liver cancer cells were harvested after treatment for 48 h, the infected cells were suspended in 500 μl of binding buffer containing 20 μl each of FITC‐Annexin V and PI according to the manufacturer's instructions (BD Bioscience, CA, USA). Then, the samples were analyzed using FACS (BD Biosciences).

### 2.5 Western blotting

Cells (5 × 10^5^) were added to 6‐well plates per well. Then, liver cancer cells were subjected to treatment using VV‐IL‐24 (4 MOI) and luteolin (5 µg/ml) or both VV‐IL‐24 (4 MOI) and luteolin (5 µg/ml). After 48 h, the cells were washed and harvested in RIPA buffer (Beyotime Institute of Biotechnology, Shanghai, China) supplemented with protease inhibitors. A Pierce™ BCA Protein Assay Agent (Thermo Fisher Scientific, Inc) was then used to measure protein levels. Samples corresponding to 20 µg protein were separated by 12% SDS‐PAGE and transferred to PVDF membrane that was blocked using 5% non‐fat dried milk for 1.5 h at room temperature. These membranes were probed with monoclonal antibodies either to IL‐24 (cat. no. 60139‐1‐Ig) or to GAPDH (cat. no. 51332) overnight at 4°C and probed with appropriate secondary antibody (cat. no. 10230269) for 2 h. To detect apoptotic genes, 10% SDS‐PAGE gels were instead used. The resultant PVDF membranes were probed using antibodies for cleaved poly‐ADP‐ribose polymerase (cat. no. 9532), XIAP (cat. no. 14334), pro caspase‐8 (cat. no. 9746), cleaved caspase‐8 (cat. no. 9748), pro caspase‐3 (cat. no. 9665), cleaved caspase‐3 (cat. no. 9664), and GAPDH was detected on the PVDF membrane and used as an internal control. Then, the membranes of cleaved PARP, PARP were probed using an appropriate rabbit secondary antibody (cat. no. 10245169). And the other membranes of XIAP, pro caspase‐8, cleaved caspase‐8, pro caspase‐3, cleaved caspase‐3, and GAPDH were incubated in the corresponding mouse secondary antibody. With the exception of the IL‐24 antibody, which was from Proteintech (Chicago, IL, USA), Cell Signaling Technology (MA, USA) provided all other antibodies. Primary and secondary antibodies were diluted 1:1,000 and 1:3,000, respectively. Protein detection was conducted with an Odyssey infrared imaging system (LI‐COR Biosciences, Lincoln, NE, USA).

### 2.6 Animal experiments

32 female BALB/c nude mice (5 weeks old) from Shanghai Slack Animal Laboratory (Shanghai, China) were housed as per the Guide for the Care and Use of Laboratory Animals (Zhejiang Sci‐Tech University).

The xenograft model was established by subcutaneously injection of 4 × 10^6^ MHCC97‐H cells into the right flank of these animals. When the transplanted tumor size was from 80‐120 mm^3^, mice were randomized into 4 groups and injected with PBS (vehicle), intratumor injection; luteolin (50 mg/kg) alone, intraperitoneal injection; VV‐IL‐24 (2 × 10^7^ plaque‐forming units) alone, intratumor injection; or VV‐IL‐24 (2 × 10^7^ plaque‐forming units), intratumor injection, together with luteolin (50 mg/kg), intraperitoneal injection. Tumor length and width were analyzed every 5 days, with tumor volume being measured as [length × width^2^/2 (mm^3^)].

### 2.7 Immunohistochemistry and histopathological staining

Tissues of the xenograft tumor, and liver, kidney, spleen tissues from each group were collected on the day 7 after the last treatment and fixed in 10% formalin for 24 h, then cut into 5 μm sections. After deparaffinization and rehydration, hematoxylin‐eosin (HE) and immunohistochemical (IHC) staining were conducted based upon standard protocols. For IHC staining, the sections were incubated with monoclonal antibodies specific for IL‐24 (1:100, Abcam), Ki‐67 (1:100, Novus Biologicals, Littleton, CO, USA), CD31 (1:100, Abcam), and cleaved caspase‐3 (1:100, Abcam). Images were taken with an IX71‐22FL/PH microscope (0.2 mm fields; Olympus Corporation, Tokyo, Japan). Magnification, ×200.

### 2.8 Statistical analysis

All experiments were repeated three times, with data given as mean ± standard deviation (SD). Student's *t* test or one‐way ANOVAs were used to analyze data with GraphPad Prism 6 (GraphPad Software, Inc, CA, USA). *p* < 0.05 was the significance threshold.

## 3 RESULTS

### 3.1 Luteolin increases the inhibitory impact of VV‐IL‐24 on liver cancer cells viability

**FIGURE 1:**
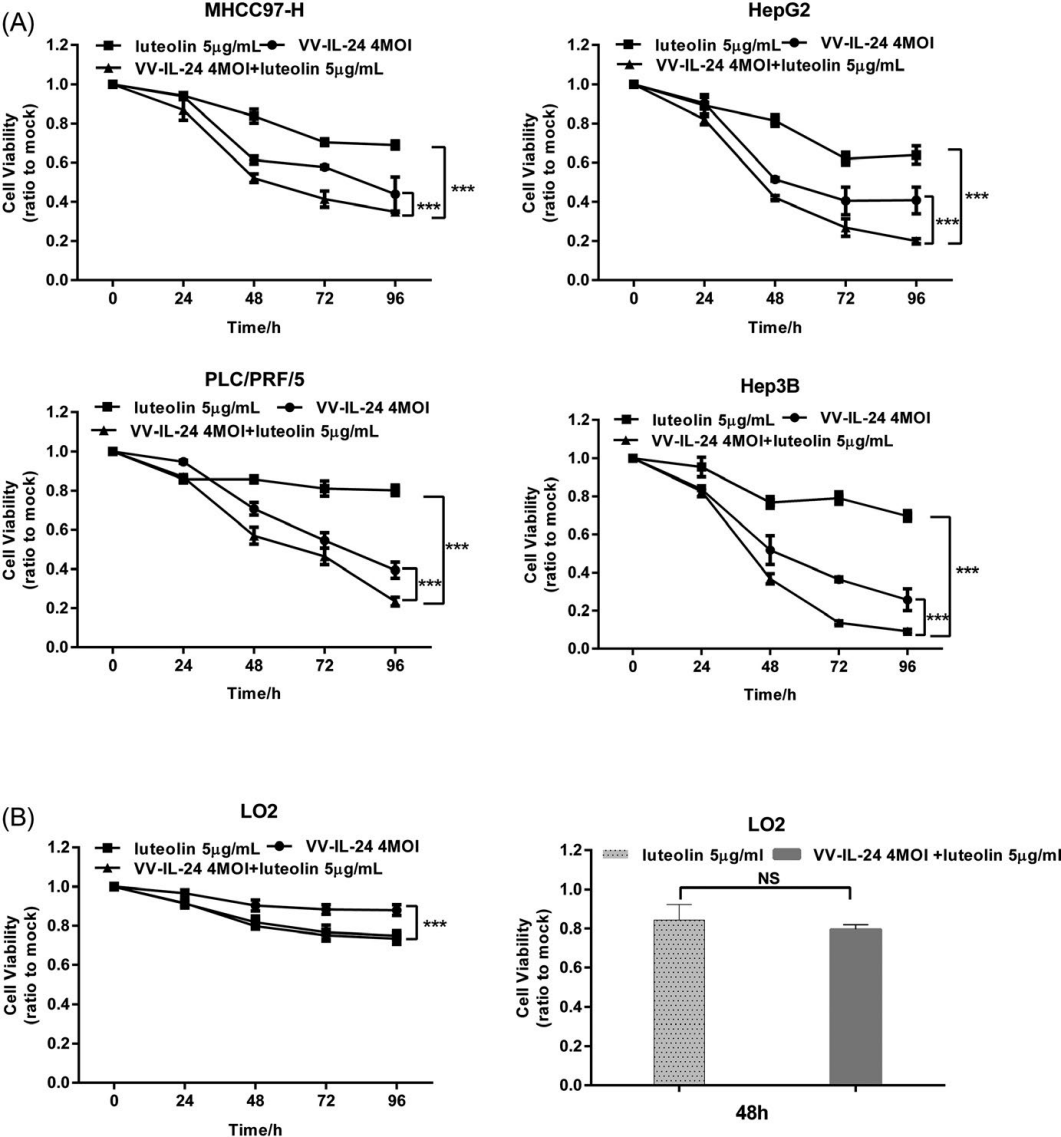
Cell viability of treated liver cancer and control cells. (A) The effect of VV‐IL‐24 (4 MOI) and luteolin (5 µg/ml) was assessed via the MTT assay in MHCC97‐H, HepG2, PLC/PRF/5, and Hep3B cells. (B) LO2 cells were subjected to treatment using luteolin (5 µg/ml) or both VV‐IL‐24 (4 MOI) and luteolin (5 µg/ml). Results are means ± standard deviation (*n* = 3). *p < 0.05; **p < 0.01; ***p < 0.001. NS, not significant; VV, vaccina virus; IL‐24, interleukin 24; MOI, multiplicity of infection

To determine whether VV‐IL‐24 (4 MOI) and luteolin (5 μg/ml) decreased cell viability more than VV‐IL‐24 (4 MOI) or luteolin (5 μg/ml) alone, the liver cancer and control cells were infected for 24 h, 48 h, 72 h, 96 h. MTT assay showed that all types of treatment reduced liver cancer cells viability in a time‐dependent fashion. In MHCC97‐H cells (Figure 1A), cell viability of combination treatment was <40% while cell viability of VV‐IL‐24 was >40% and cell viability of luteolin was >60% at 96 h. In HepG2 cells (Figure 1A), cell viability of combination treatment was <25% while cell viability of VV‐IL‐24 was >40% and cell viability of luteolin was >60% at 96 h. In PLC/PRF/5 cells (Figure 1A), cell viability of combination treatment was <30% while cell viability of VV‐IL‐24 was >30% and cell viability of luteolin was >65% at 96 h. In Hep3B cells (Figure 1A), cell viability of combination treatment was <15% while cell viability of VV‐IL‐24 was >20% and cell viability of luteolin was >60% at 96 h. Notably, treatment with VV‐IL‐24 and luteolin was similar to luteolin alone in normal LO2 cells (Figure 1B) at 48 h, which suggested that VV‐IL‐24 plus luteolin have less toxic impact to normal cells.

The data suggested that the liver cancer cells viability of VV‐IL‐24 was obviously lower than that of luteolin, and the combination of VV‐IL‐24 with luteolin was significantly inhibited liver cancer cells viability than VV‐IL‐24 or luteolin alone and did not affect the proliferation of the normal liver cells LO2.

### 3.2 VV‐IL‐24 and luteolin act synergistically on liver cancer cells

**FIGURE 2:**
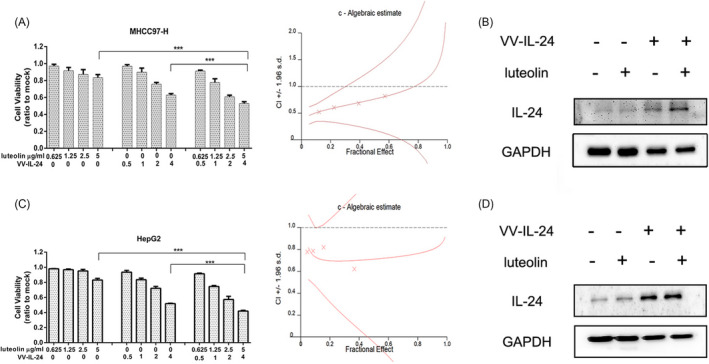
Luteolin promotes VV‐IL‐24 mediated transgene expression in liver cancer cells. (A) MTT assays were used to measure MHCC97‐H cell viability, with CalcuSyn being used to quantify data and with CI values being indicated by X‐marks. (B) IL‐24 levels in MHCC97‐H cells were assessed by Western blotting. (C) HepG2 cell viability was examined with an MTT assay and quantitated as in (A). (D) IL‐24 levels were analyzed in HepG2 cells by Western blotting. Results are means ± standard deviation (*n* = 3). *p < 0.05; **p < 0.01; ***p < 0.001. VV, vaccinia virus; IL‐24, interleukin 24; MOI, multiplicity of infection; CI, combination index

To establish whether VV‐IL‐24 combined with luteolin has a synergistic effect, liver cancer cells were treated with VV‐IL‐24 (0.5 MOI, 1 MOI, 2 MOI, 4 MOI), luteolin (0.625 μg/ml, 1.25 μg/ml, 2.5 μg/ml, 5 μg/ml), or both at a fixed 4:5 proportion for 48 h. The combination index (CI) values were served as X‐marks on the graph. In MHCC97‐H (Figure 2A) and HepG2 (Figure 2C) cells, the CI values of all the data were <1, indicating that the combination of VV‐IL‐24 and luteolin had a synergistic effect in liver cancer cells.

To determine whether luteolin can enhance oncolytic vaccinia virus VV‐IL‐24 mediated gene expression, the IL‐24 protein in treated liver cancer cells was assessed via Western blot. Liver cancer cells were treated using VV‐IL‐24 (4 MOI), luteolin (5 μg/ml), or both VV‐IL‐24 (4 MOI) and luteolin (5 μg/ml). In MHCC97‐H (Figure 2B) and HepG2 (Figure 2D) cells, the combination treatment of VV‐IL‐24 and luteolin upregulated IL‐24 gene expression more than VV‐IL‐24 or luteolin alone, suggesting that luteolin could increase VV‐IL‐24 expression in liver cancer cells.

### 3.3 VV‐IL‐24 and luteolin enhance the liver cancer cells apoptosis

**FIGURE 3:**
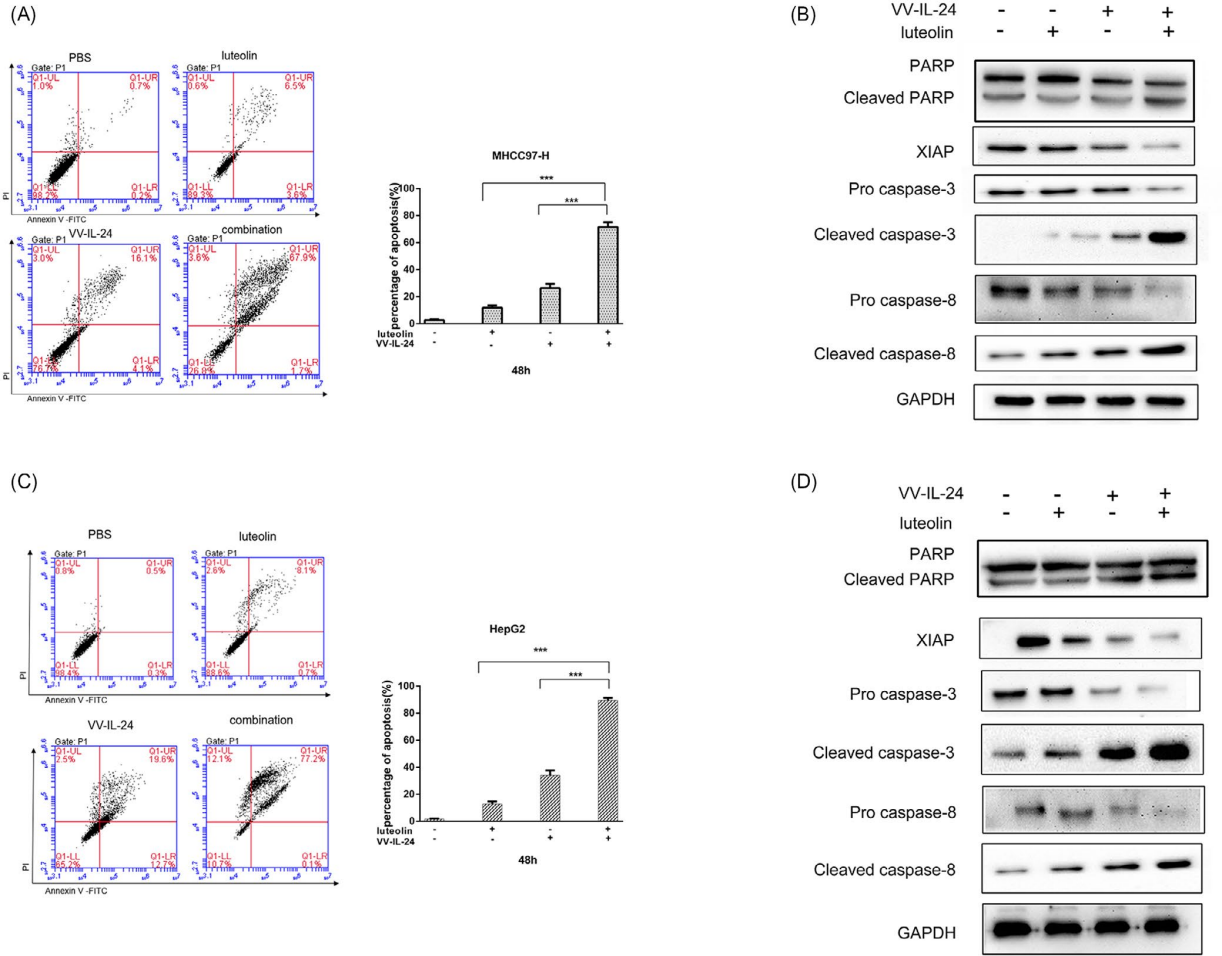
The combination of VV‐IL‐24 and luteolin induces apoptosis in liver cancer cells. (A) The percentage of apoptotic cells in MHCC97‐H cells was analyzed via flow cytometric analysis. (B) Apoptosis‐associated protein expression in MHCC97‐H cells was assessed via Western blotting. (C) The percentage of apoptotic cells in HepG2 cells was detected using flow cytometric analysis. (D) The expression of apoptosis‐associated protein in HepG2 cells was detected by Western blotting. The cells were treated with VV‐IL‐24 (4 MOI), luteolin (5 µg/ml) and the combination of VV‐IL‐24 (4 MOI) plus luteolin (5 µg/ml). GAPDH was used as a loading control. Results are means ± standard deviation (*n* = 3). *p < 0.05; **p < 0.01; ***p < 0.001. XIAP, E3 ubiquitin‐protein ligase; PARP, poly‐ADP‐ribose polymerase; VV, vaccina virus; IL‐24, interleukin IL‐24; MOI, multiplicity of infection

To explore whether the antitumor properties of the combination treatment were due to an increase in cell death, flow cytometry measurements of apoptosis were performed. In MHCC97‐H cells (Figure 3A), the treatment by VV‐IL‐24 and luteolin resulted in a markedly higher fraction of apoptotic, 62%, than treatment with only VV‐IL‐24, treatment with VV‐IL‐24, 36%, or only luteolin, 10%. And in HepG2 cells (Figure 3C), the test of cell death by flow cytometry displayed that compared with 11% for luteolin and 39% for VV‐IL‐24, treatment with VV‐IL‐24 and luteolin resulted in an obviously improvement percentage of apoptotic cells to 75%. Both MHCC97‐H and HepG2 cells treated with combined VV‐IL‐24 and luteolin had a significantly higher percentage of apoptosis relative to either individual treatment (*p* < 0.001). Therefore, combination therapy induced more apoptosis compared with any single treatment in liver cancer cells.

We also examined the important caspase‐dependent apoptotic signaling protein expression by Western blotting. Increased expression of cleaved PARP, cleaved caspase‐3, cleaved caspase‐8 and reduced procaspase‐3 and procaspase‐8, XIAP expression levels were significantly detected in combination‐treated MHCC97‐H (Figure 3B) and HepG2 cells (Figure 3D). We have quantified these blots of Figure 3B,D. The expression of cleaved PARP was the highest in the combination group. These data indicated that combination treatment induced more cellular apoptosis than in VV‐IL‐24 or in luteolin treatment alone via activation of caspase‐dependent apoptotic signaling pathway.

### 3.4 VV‐IL‐24 and luteolin synergistically inhibit the progression of liver cancer tumor xenograft

**FIGURE 4:**
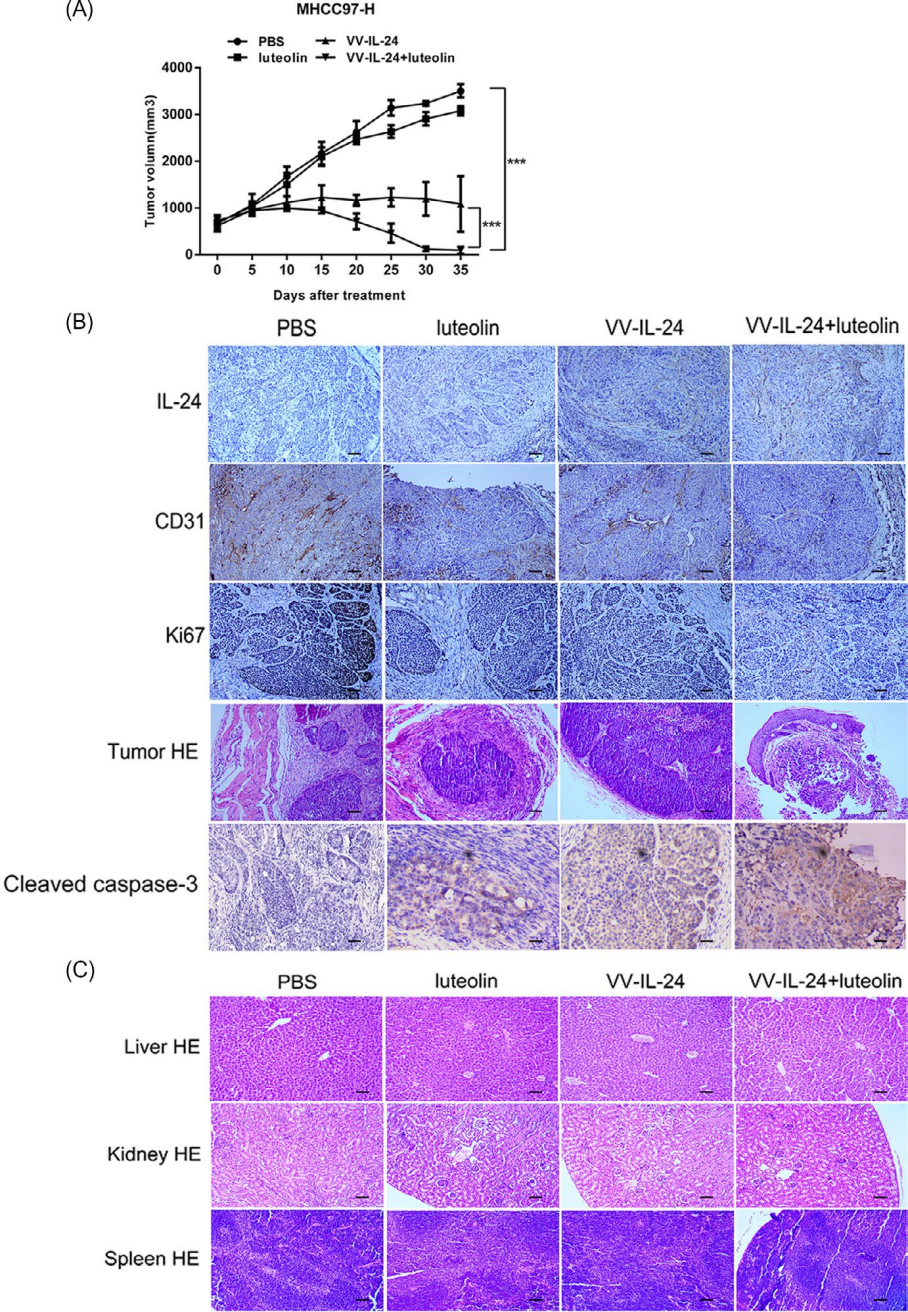
VV‐IL‐24 and luteolin synergistically inhibit MHCC97‐H cell growth in a mouse xenograft model. (A) Tumor dimensions were measured every 5 days and the volume was computed as (length × width^2^/2 [mm^3^]). Results are means ± standard deviation (*n* = 6). ***p < 0.001. (B) The expression of IL‐24, CD31, Ki67 and cleaved caspase‐3 in tumor tissues was detected by IHC, and the areas of cellular necrosis within the tumors were examined by the HE staining. (C) Toxic effects in the liver, kidney and spleen tissues were examined by HE staining in each group. Enlargement, ×200. Bar: 50 µm. VV, vaccina virus; IL‐24, interleukin 24; IHC, immunohistochemistry; HE, hematoxylin and eosin

To assess the antitumor efficacy of the combination therapy in vivo, the MHCC97‐H xenograft mouse model was established. Tumor growth curves demonstrated that xenograft MHCC97‐H tumor growth was obviously impaired in the combination treatment group of VV‐IL‐24 and luteolin than in single treatment group on day 15 of treatment (Figure 4A). And on day 35, the tumor volume in the groups treated with PBS reached 3,503 mm^3^, with luteolin 3,080 mm^3^, and with VV‐IL‐24 1,088 mm^3^, while in the combination group of VV‐IL‐24 with luteolin tumor volume was 105 mm^3^, documenting significant inhibition of the tumor growth (Figure 4A). Thus, the combination treatment had a greater antitumor effect in vivo than treatment with VV‐IL‐24 or luteolin alone. And the data indicated that VV‐IL‐24 and luteolin acted synergistically to inhibit the growth of liver cancer tumor xenografts.

On day 7 after the last treatment, a mouse was randomly selected from each group. Immunohistochemical (IHC) staining (Figure 4B) demonstrated that after the treatment with the combination of VV‐IL‐24 and luteolin, the IL‐24 protein was expressed in tumor tissue at a markedly higher level than in the other groups. CD31, a vascular endothelial marker, is associated with the tumor of development, angiogenesis, and metastasis. And Ki67 is a proliferative cell‐associated nuclear antigen of a variety of tumors. IHC revealed that CD31 and Ki67 staining was significantly weaker in the combination treatment group relative to treatment with just VV‐IL‐24 or luteolin (Figure 4B). We have added the expression of cleaved caspase‐3 by IHC staining (Figure 4B). The results confirmed that protein level of cleaved caspase‐3 increased obviously in VV‐IL‐24 and luteolin combination treatment group than in VV‐IL‐24 group or in luteolin group, which proved that the induction of cleaved caspase‐3 may contribute to the enhancement antitumor efficacy of VV‐IL‐24 and luteolin in vivo. And these finding also show that VV‐IL‐24 combined with luteolin which had a stronger efficacy in vivo may contributed to the increased IL‐24 gene expression and the inhibition of tumor cells proliferation and angiogenesis.

Hematoxylin‐eosin (HE) staining showed that the combined VV‐IL‐24 and luteolin treatment resulted in a more severe cytopathic effect in tumor tissues than the treatment with VV‐IL‐24 or luteolin alone (Figure 4B). In addition, the observation that the liver, kidney and spleen tissues of the combination group had no or little cell damage demonstrated that VV‐IL‐24 and luteolin had no or little toxic effects occurred on these tissues (Figure 4C).

## 4 DISCUSSION

Many studies have demonstrated that luteolin is a key inhibitor of tumor cell proliferation that can induce apoptotic death, as shown in human myeloid leukemia ^21^ and melanoma cells.^22^ Proliferation and chemo‐resistance of the liver cancer cell can occur as a consequence of genetic and epigenetic changes, and all of these alterations can serve as potential therapeutic targets.^23^, ^24^, ^25^ Optimal approaches to establishing efficient antitumor responses without significant toxicity are vital for liver cancer.^26^, ^27^, ^28^ A strategy involving a combination of VV‐IL‐24 and luteolin that could effectively suppress tumor growth or decrease toxicity may provide a potentially promising treatment for liver cancer compared with standard treatments such as chemotherapy, radiation therapy and surgical resection.

Cancer‐targeting gene‐viro‐therapy (CTGVT), which employs oncolytic viral vectors encoding anticancer genes, is associated with potent anticancer activity. A large number of studies have shown vaccinia virus to be well suited to biological cancer therapies.^29^, ^30^, ^31^ It is worth noting that the combination of the oncolytic virus and chemotherapy, immunotherapy or other oncolytic vaccine viruses can utilize additional mechanisms of action to augment antitumor efficacy.

The present investigation demonstrated that oncolytic VV harboring the IL‐24 gene, VV‐IL‐24, efficiently inhibited liver cancer cells viability. Furthermore, VV‐IL‐24 combined with luteolin triggered a higher magnitude of liver cancer cell apoptosis than VV‐IL‐24 or luteolin alone by flow cytometry assay.

Apoptosis, autophagic cell death and necrosis are programmed cell death pathways.^32^ Inducing more apoptosis of cancer cells is essential for cancer treatment. The cleavage of PARP is considered to be an important indicator of cellular apoptosis and is generally considered to be an activation indicator of cleavage caspase‐3. Our results indicated that the combination VV‐IL‐24 and luteolin treatment induced more cleavage PARP and cleaved caspase‐3 than the other groups in MHCC97‐H and HepG2 cells, which resulted in a greater degree apoptosis of combination therapy. Furthermore, the decreased expression of XIAP after the combination treatment compared with other treatments significantly increased the effect of apoptosis in both of these cells. However, whether ROS contributes to the efficacy of combination therapy remains to be explored.

Most importantly, the combination of VV‐IL‐24 and luteolin was significantly more effective as a suppressor of tumor growth in the MHCC97‐H nude mouse xenograft model than luteolin or VV‐IL‐24 alone. Since both luteolin and IL‐24 have been shown to inhibit tumor growth through the JNK signaling pathway, whether the combination therapy can enhance the activation of the JNK signaling pathway still needs further confirmation. IHC showed that the expression of CD31 and Ki67 was lower following the combination treatment relative to single agent treatment, suggesting that the combination therapy inhibits liver cancer cells proliferation and angiogenesis. Moreover, the combination of VV‐IL‐24 and luteolin produced more extensive cell death in the tumor tissue than treatments with single components, while having no or little toxic effects on the liver, kidney, and spleen tissues.

Recent studies have shown MDA‐7/IL‐24 to control many microRNAs, such as miR‐221, which is upregulated in many types of cancer.^33^ MDA‐7/IL‐24 downregulated miR‐221, which in turn induces Beclin‐1, leading to autophagy. IL‐24 has been shown to promote the populations of CD4^+^ and CD8^+^ T cells in diverse cancer models.^34^, ^35^ In our study, luteolin can increase VV‐mediated IL‐24 gene expression in liver cancer cells in vitro and in vivo. The ERK signaling pathway is necessary for the replication of vaccinia virus,^29^ and whether luteolin promotes the expression of IL‐24 through the ERK signaling pathway needs to be further explored. Furthermore, the combination therapy that stimulates liver cells autophagy and regulates the immune system should also be verified in the future.

## CONFLICT OF INTEREST

The authors have declared no competing interest for this work.

## AUTHORS' CONTRIBUTIONS

Yigang Wang conceived and designed the experiments; Chunming Wang, Qiang Li, Boduan Xiao and Huiling Fang analyzed the data; Fang Huang performed the histological examination of animal experiment; Chunming Wang wrote the paper; Yigang Wang and Biao Huang revised the paper. All authors read and approved the final manuscript.

## CONSENT FOR PUBLICATION

The authors have confirmed that all details can be published and prepared to provide copies upon reasonable request.

## Data Availability

The datasets used and/or analyzed during the current study are available from the corresponding author upon reasonable request.
